# Multicentric Castleman’s disease in HIV/AIDS patients at an urban HIV clinic in Atlanta, Georgia, in the combined antiretroviral therapy era

**DOI:** 10.1186/1750-9378-7-S1-P20

**Published:** 2012-04-19

**Authors:** Rathi N Pillai, Clifford Gunthel, Marylyn Adamski, Marina Mosunjac, Minh Ly Nguyen

**Affiliations:** 1 Winship Cancer Institute, Emory University, Atlanta, GA, USA; 2 Division of Infectious Diseases, Emory University School of Medicine, Atlanta, GA, USA; 3 Infectious Disease Program, Grady Health System, Atlanta, GA, USA; 4 Department of Pathology, Emory University School of Medicine, Atlanta, GA, USA

## Background

Multicentric Castleman’s disease (MCD), which has been associated with human herpesvirus-8 (HHV-8), is a lymphoproliferative disorder with an increased prevalence in HIV positive patients [1]. We describe our experience with MCD in a group of patients with HIV/AIDS in an urban HIV clinic.

## Methods

Our clinic serves annually 5,000 patients diagnosed with AIDS. Patients with a diagnosis of multicentric Castleman’s disease between 2006 and 2010 were identified from the pathology database at Grady Memorial Hospital or referrals to the clinic. Clinic charts and medical records were abstracted. Patients’ demographics, CD4 counts, HIV viral load, HIV and MCD treatment and outcomes were recorded.

## Results

Nine patients diagnosed with MCD were identified in our HIV/AIDS population. All patients were male and reported sex with men (MSM) as their risk for HIV infection. The mean age at MCD diagnosis was 39.22 ± 11.40; the mean CD4 cell count nadir was 68.33 ± 62.2 cells/mm^3^. 85% (7/9) were on cART (combined antiretroviral therapy) at the time of MCD diagnosis with a mean CD4 count of 233.67±157.44 cells/mm^3^. MCD was of the hyaline vascular variant in 3 patients, plasma cell variant in 2, transitional in 1 patient, and unspecified in 2 patients. Systemic symptoms were present in three patients. Five patients had both Kaposi sarcoma (KS) and MCD (2 with KS occurring after MCD diagnosis, 1 with KS before MCD, 2 with KS and MCD diagnosed simultaneously). Most of the patients were anemic with mean hemoglobin of 8.99±4.04 g/dL and hypoalbuminemic (2.31±0.96). 85% had anemia, hepatosplenomegaly, and low albumin at diagnosis. Treatment consisted of valgancyclovir, chemotherapy and/or rituximab. In the 5 patients who died, the mean time from MCD diagnosis was 425.2±447 days.

## Conclusions

HIV-associated MCD is characterized by lymphadenopathy, splenomegaly, anemia and hypoalbuminemia. Among the diseases associated with HHV8 (KS, primary effusion lymphoma, and MCD), MCD appears to be the least affected by cART use or degree of immunosuppression [2]. In our cohort, 85% of patients had a CD4 count above 200 at MCD diagnosis.The survival with cART is still dismal, with one year survival of 50%. Larger multicenter study is needed to better understand the pathogenesis of HIV-associated MCD and its treatment.
